# Coherent power amplification of third-order harmonic femtosecond pulses at thin-film up-conversion nanoparticles

**DOI:** 10.1038/s41598-019-41591-6

**Published:** 2019-03-25

**Authors:** Yi Gao, Hyub Lee, Wen Xu, Jiannan Jiao, Peng Chen, Dong-Hwan Kim, Young-Jin Kim

**Affiliations:** 1 School of Mechanical and Aerospace Engineering, Nanyang Technological University, 50 Nanyang Avenue, Singapore, 639798 Singapore; 2 School of Chemical and Biomedical Engineering, Nanyang Technological University, 70 Nanyang Drive, Singapore, 637457 Singapore; 3 School of Chemical Engineering, Sungkyunkwan University, 2066, Seobu-ro, Jangan-gu, Suwon-si, Gyeonggi-do, 16419 Republic of Korea

## Abstract

Third harmonic generation (THG) is a nonlinear optical process attractive in high-resolution interfacial studies, sub-wavelength light manipulation, and bio-molecular detection due to its capability of converting low-energy quanta into a quantum of a higher energy. One of the limitations in utilizing THG is its low power conversion efficiency; thus, various THG enhancement methods have been researched by involving plasmonic coupling effects or utilizing electric band gap resonances at quantum dots or two-dimensional materials. Meanwhile, lanthanide ion-doped up-conversion nanoparticles (UCNPs) can be excited by a multi-photon process similar to THG, but its interaction or resonance with THG has not been studied to date. In this Communication, we demonstrate the first coherent amplification of third-order harmonic femtosecond pulses at multi-layered UCNP thin-film with an amplification factor of 7.8. This amplification is made by the resonance interaction of incident femtosecond laser field, generated third-order harmonics, and the electric band gaps of UCNPs. The power contribution of the third-order harmonic and the up-conversion luminescence (UCL) is strongly dependent on the sample geometry due to the reabsorption effect. For in-depth understanding of the emission characteristics, spectral-domain, time-domain, radio-frequency (RF) domain, and polarization-dependence analysis were addressed. This coherent amplification of third harmonic (TH) at UCNP thin-films enables us to attain higher power, shorter wavelength, and ultra-short femtosecond pulses generated from a simple thin-film structure near to the target samples, which will pave a way to an ultrafast short-wavelength laser platform for material characterization, sub-wavelength photonics, and biomolecular detection.

## Introduction

Nonlinear optics describes the phenomena occurring when intense light passes through an optical medium, where photons of new optical frequencies are coherently generated^1^. Two of the simplest and most representative nonlinear optical processes are second harmonic generation (SHG) and third harmonic generation (THG), which doubles and triples the input photon frequencies^2^. While even-order nonlinear processes including SHG require the optical materials to have non-centrosymmetric atomic structures, odd-order nonlinear processes including THG are more general, so can be observed in most of optical medium due to their relaxed symmetry rules^2^. Therefore, THG has attracted significant interests as a coherent optical frequency conversion process in diverse research fields. At macroscale, it has been intensively exploited for coherent up-conversion of tunable laser light^3^ and also for label-free imaging of biological samples^4,5^. At micro- and nano-scales, THG shows enormous potentials in sub-diffraction-limit optical imaging^6^ and surmounting the Shockley–Queisser efficiency limit in single-junction solar cells^7^. Despite these outstanding virtues, achieving high efficiencies in harmonic conversion process has remained a great challenge. Extensive researches have been made for improving THG efficiencies by adopting plasmonic coupling at noble metal nanoantennas^8,9^, plasmonic Fano structures^10^, and plasmonic metamaterials^11^. However, even with three-orders of magnitude field enhancement, the overall emission harmonic intensity (total number of photons) from these plasmonic structures have been still limited because the local field enhancement in metallic plasmonic structures is confined to an extremely small volume^12–14^. By using dielectric nanoparticles having higher refractive indices, a larger mode volume can be attained, but at the expense of weaker field enhancement, which will still limit the THG’s amplification factor^15,16^. Although successful, the expensive and time-consuming manufacturing processes of plasmonic nanostructures, such as e-beam lithography, increase weight, complexity, and cost to the optical system.

Electric band gap resonance has been also considered as another power enhancement mechanism for the nonlinear harmonics. Quantum dots^17^ and two-dimensional transition metal dichalcogenides^18^ have been reported to be capable of enhancing the power conversion efficiency of nonlinear harmonic generation. The resonance between the electric band gap of quantum dots and nonlinear harmonics enables the inter-sub-band transition of the electrons within the quantum well, which greatly enhances the nonlinear susceptibility^19^. Meanwhile, when the energy band gap between the electrons and holes, which is known as the exciton, in two-dimensional transition metal dichalcogenides is resonant with the photon energy of the incident laser beam, the effective mass of the electrons can be increased thanks to the strong quantum confinement, which results in stronger nonlinear optical harmonic generation^18^. However, the resonance among three bodies, the incident laser field, TH, and electric energy gap, has remained unexplored to date; up-conversion nanoparticle (UCNP) can be a promising material candidate for achieving this three-body resonance. By doping lanthanide ions (e.g. Er^3+^) into hosting crystalline matrices (e.g. NaYF_4_) with proper concentration, nanoparticles can be formed with stable ladder-like energy states, which enables the sequential absorption of multiple photons in the near-infrared (NIR) wavelength and subsequent light luminescence over the visible and ultraviolet (UV) wavelengths. Unlike TH, the UCNP luminescence comes from real energy states instead of the nonlinear optical dipoles; thus, UCL is generally not coherent with the incidence laser field so has a much longer lifetime than TH^20^. To date, the mutual interrelation of THG and UCNP luminescence has not been studied.

In this report, we demonstrate the power amplification of third-order harmonic femtosecond laser pulses at self-assembled thin-film NaYF_4_:Er^3+^ UCNPs. Nano-sized UCNPs were used in the experiment because the quantum efficiency of UCNPs are usually higher than bulk up-conversion materials due to better host material crystallinity and more uniform distribution of lanthanide ions^20^. Besides, THG is a pump-power-dependent process; therefore, thinner up-conversion films are beneficial to minimize the unexpected absorption of incident laser beam. While a thin up-conversion film of nanometer thickness has been difficult to fabricate using bulk materials, it can be easily self-assembled from nano-sized UCNPs.By focusing NIR femtosecond pulses onto ~80 nm thick thin-film UCNPs, the resonance of the incident laser field, its TH, and the electric energy gap of UCNPs was made, which enabled a 7.8-times stronger TH emission. This is the first report on coherent power amplification of nonlinear harmonics in UCNPs, to the best of our knowledge. The amplification factor was strongly dependent on the sample geometry; depending on thin-film position, whether the film is heading to or against the incident laser beam, the THG emission yield was changed from 2.5 to 3.5, which implies that UCL plays a crucial role in TH amplification. For in-depth understanding of the amplified pulses, spectral-domain, time-domain, radio-frequency (RF) domain, and polarization-dependence tests have been performed. These tests conclude that the TH wavelength, repetition rate, pulse duration, and polarization state of the incident laser field are well maintained throughout this novel amplification process at thin-film UCNPs. Besides, self-assembled thin-film of chemically synthesized UCNPs is much cheaper and faster to fabricate compared to the plasmonic nanostructures manufactured by lithography, which makes it suitable for mass production for wider applications.

## Results and Discussion

### Power amplification of TH at thin-film UCNPs: system layout and sample preparation

**Figure 1:**
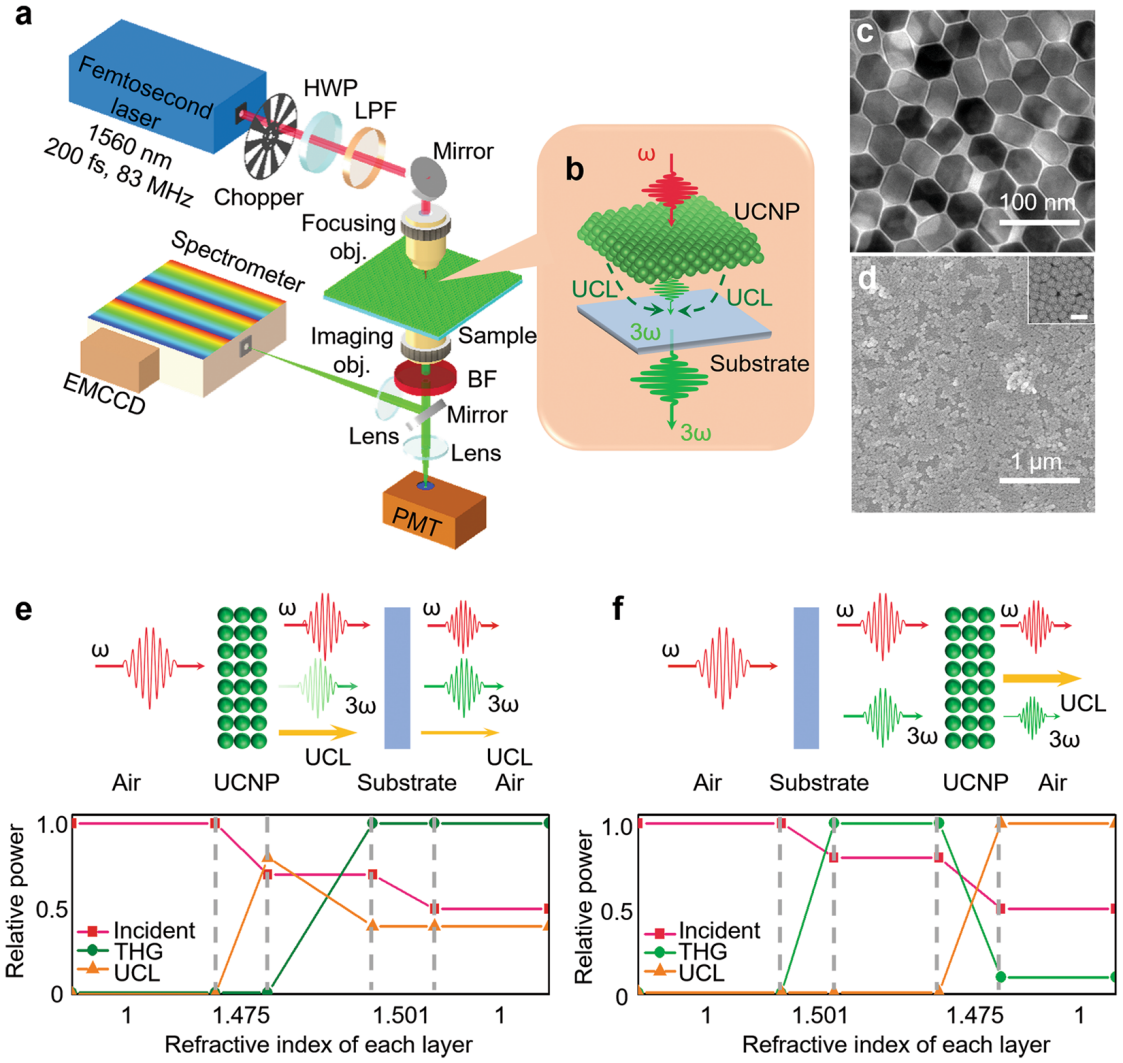
Power amplification of third-harmonic femtosecond laser pulses at a self-assembled thin-film UCNPs. (**a**) Optical system. (**b**) Amplified ultrafast TH at thin-film UCNPs. (**c**) TEM image of synthesized UCNPs. (**d**) SEM image for a self-assembled UCNP thin-film; the inset shows a magnified view of the UCNP thin-film. The scale bar for the inset corresponds to 50 nm. Optical schematics and relative power distributions among the incident laser, its TH, and UCL across the sample at (**e**) laser-UCNP-substrate and (**f**) laser-substrate-UCNP geometries. Abbreviations: UCL: up-conversion luminescence, HWP: half-wave plate, LPF: long-pass filter, obj: objective lens, BF: bandpass filter PMT: photomultiplier tube and EMCCD: electron multiplying charge-coupled device.

Figure 1 illustrates the experimental setup for power amplification of TH at thin-film UCNPs. A mode-locked Er-doped fiber femtosecond laser (Toptica FemtoFiber pro NIR) was used as the excitation light source with a central wavelength of 1,560 nm, pulse duration of 200 fs, and repetition rate of 83.3 MHz. The multi-layered thin-film UCNPs were deposited on top of two substrates, a borosilicate glass coverslip (in sections 2, 3, 4 and 6), and a crystalline intrinsic (100) silicon (Si) wafer (in sections 5 and 6). A half-wave plate (Thorlabs WPH10M-1550) was utilized to control the polarization state of the excitation laser beam. The SH and TH generated from the femtosecond fiber laser and power amplifiers were removed by a 1,200 nm long-pass filter (Thorlabs FEL-1200). The excitation beam was focused onto the interface between substrate and thin-film UCNPs by a 40× objective lens (Newport LI-40X) of a 0.65 numerical aperture (NA); the resulting output TH and UCL were collected by another 40× objective lens (Olympus UPlan 40X) with a 0.65 NA. The collected light was then delivered to a broadband visible spectrometer (Andor’s Shamrock 193i) in combination with an electron multiplying charge coupled device (EMCCD; Andor’s IXon Ultra) for the spectral-domain analysis and a photomultiplier tube (PMT) (Thorlabs PMT1001M) for the time- and frequency-domain analysis. A series of optical band-limiting filters (Thorlabs FES-550 and FEL-600) were utilized with the PMT when measuring the rise and decay time of green or red emissions from the sample.

NaYF_4_:Er^3+^ UCNPs were chemically synthesized following the procedures reported by Chen *et al*.^20^. The self-assembled UCNP thin-film was then fabricated by the interfacial assembly method^21^. Figure 1c,d show the synthesized NaYF_4_:Er^3+^ UCNPs and the self-assembled UCNP thin-film measured by a transmission electron microscope (TEM) and a scanning electron microscope (SEM). The mode-locked femtosecond laser pulses were tightly focused onto the UCNP-substrate interface. The resulting beam diameter was ~7.6 μm and the power density at the focal spot was ~4.4 × 10^9^ W·cm^−2^. As a surface nonlinear phenomenon, the THG at the interface between the substrate and UCNP is significantly stronger than the bulk body by more than 10^5^ times^1^. Due to this high peak power of the femtosecond pulse laser, both THG and UCL signals were clearly observed. The TH and UCL had a good spectral overlap at ~536 nm, which enabled the efficient power amplification (See Fig. 1b). Interestingly, TH amplification factor was found to be strongly dependent on the excitation sample geometry. When the UCNP thin-film is placed facing towards the excitation laser beam (laser-UCNP-substrate geometry as shown in Fig. 1e), UCL gets strongly coupled to TH, resulting in TH yield enhancement. When UCNP thin-film is placed facing against the excitation laser beam (laser-substrate-UCNP geometry as shown in Fig. 1f), TH at 520 nm is generated firstly at the substrate-UCNP interface, then passes through the UCNP thin-film, which leads to reabsorption of TH by Er^3+^ ions in UCNPs^22^. This reabsorption does not occur in the laser-UCNP-substrate geometry since the propagation direction of TH remains the same with fundamental laser beam^1^. While the absorption peak of Er^3+^ at 1,560 nm significantly enhances the absorption of fundamental laser beam at the interface between UCNP thin-film and substrate^19^, the nonlinear optical dipoles at the UCNP-substrate interface are in resonance with the energy gap between the excited states and ground levels, which triggers the energy transfer from the excited electrons to TH^17^. This effect results in the power amplification of TH and, in turn, the power reduction of UCL. Meanwhile, in laser-substrate-UCNP geometry, TH is firstly generated at the substrate-UCNP interface and propagates along with the fundamental laser beam; here, the absorption peak of Er^3+^ at 520 nm^22^ allows for the reabsorption of TH at the UCNP thin-film, which leads to less efficient TH amplification. The reabsorbed TH then acts as secondary excitation source for UCNP thin-film, resulting in a higher intensity at UCL. The refractive indices of different layers are shown in the x-axis of Fig. 1(e); UCNP has a refractive index closer to substrate, which proves that the higher THG intensity does not come from the refractive index difference between the substrate and thin-film UCNP. Two substrates were utilized in this investigation, a fused silica glass coverslip and a crystalline intrinsic (100) Si wafer.

### Spectral domain analysis of the output emission from thin-film UCNPs

**Figure 2:**
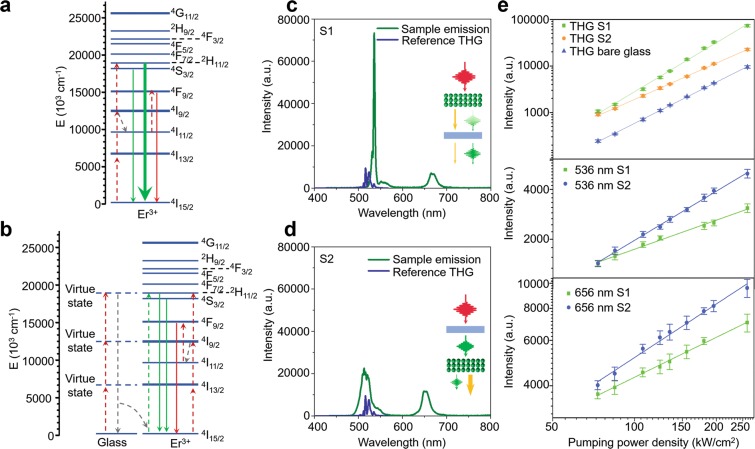
TH and UCL emissions in two different geometries. (**a**,**b**) Energy diagrams at laser-UCNP-substrate and laser-substrate-UCNP geometries. (**c**,**d**) Representative spectra for sample emission and reference THG from bare glass for laser-UCNP-substrate and laser-substrate-UCNP geometries. (**e**) Emission intensity vs pumping power density plot for THG at 536 nm, UCL at 536 nm, and UCL at 661 nm. S1 and S2 stand for the laser-UCNP-substrate and laser-substrate-UCNP geometries, respectively.

Figure 2a shows the energy diagram of NaYF_4_:Er^3+^ UCNPs. The one-third of the central wavelength of the femtosecond laser matches well with one of the UCNP emission peaks, formed by the electron relaxation from ^4^H_11/2_ to ^4^I_15/2_. This enables the resonance of the incident NIR femtosecond laser (working as the fundamental pump light), its TH (working as the seed light), and UCL (working as the secondary pump light). The emission spectra from two different sample geometries are shown in Fig. 2c,d. The emission from the laser-UCNP-substrate geometry contains a narrow peak at 536 nm with a 3.7 nm bandwidth and two relatively broader UCL peaks at 551 and 661 nm. The narrower THG peak at 536 nm here matches well with one of the UCNP emission peaks, made by the electron relaxation from ^4^H_11/2_ to ^4^I_15/2_. Compared to the reference THG measured with a bare glass substrate without the UCNP thin-film (See the blue curve in Fig. 2c,d), the spectral intensity ratio of the two peaks, one at 536 nm and the other at 516 nm is 4.9-times higher than the ratio in the laser-UCNP-substrate geometry, suggesting more efficient THG amplification at 536 nm while maintaining the original spectral bandwidth of TH due to its better match to energy band gap between ^4^H_11/2_ to ^4^I_15/2_. The detailed TH pulse characteristics will be verified further in subsequent sections. This power amplification mainly comes from the resonance between the ^4^H_11/2_-^4^I_15/2_ energy gap and the nonlinear electron dipole oscillation at the UCNP-substrate interface excited by the femtosecond pulse laser^23,24^, evidenced by the wavelength-selectivity of UCL-based TH enhancement. The amplification mechanism was further evaluated by the emission yield calculated from the linear-fit slope of the emission-intensity vs. pump-power-density plot as shown in Fig. 2e. A 7.8-times higher TH intensity was detected at 536 nm from the sample with UCNP thin-film compared to bare glass slide, which leads to a normalized amplification efficiency of 35,000% *W*^−1^ *cm*^−2^ ^25^. The conversion efficiency of THG was estimated using a photomultiplier tube (PMT; Thorlabs, PMT1001M) to be 3.6 × 10^−8^ for laser-UCNP-substrate geometry, 1.1 × 10^−8^ for laser-substrate-UCNP geometry, and 4.7 × 10^−9^ for reference glass slide. The THG emission yield from the reference bare glass slide was 2.85, while the emission yield from the UCNP thin-film reaches 3.51, which is higher than the predicted value by the power dependence law for nonlinear perturbative harmonic generation (*I*_*THG*_ ∝ *P*_*n*_, where *n* is 3 in THG). Meanwhile, the linear-fit slopes for UCNP emissions at 551 nm and 664 nm were 0.51 and 0.57, respectively; these are far lower than 3 (which is the case for THG) due to the strong scattering of UCL, high beam divergence due to the low coherence, and the limited collection angle of the imaging objective lens, and agree well with the previous report^22^. The electrons of Er^3+^ are excited to ^2^H_11/2_ following the three-step excitation process through ^4^I_13/2_ and ^4^I_9/2_. Due to the resonance between TH and ^2^H_11/2_-^4^I_15/2_ energy gap, the major portion of the electrons in ^2^H_11/2_ move through the radiative pathway and enhance THG process, while the minor part of the electrons decay non-radiatively to ^4^S_3/2_ for further relaxation to the ground state, resulting in the minor peak at 551 nm. At ^4^I_9/2_, some part of the electrons also decays non-radiatively to ^4^I_11/2_ due to the relatively higher stability of ^4^I_11/2_^20^; then, these electrons are pumped to ^4^F_9/2_, followed by relaxation to the ground state so emit photons at 661 nm.

In the laser-substrate-UCNP geometry, TH was also excited at the glass-UCNP interface. However, due to the absorption peak of Er^3+^ ions located at 520 nm^22^, the generated TH are reabsorbed by UCNP multi-layers and utilized to pump Er^3+^ electrons to ^2^H_11/2_; this results in weaker and broader TH spectrum as shown in Fig. 2d. Due to the presence of the board UCL, the detected spectrum at the green region is wider than the reference TH. In Fig. 2e, the intensity of THG at highest pumping power was 69% lower than laser-UCNP-substrate geometry, while UCL at 551 nm and 661 nm was 1.6- and 1.3-time higher. The linear-fit slopes are 2.59 for the THG peak at 536 nm, 0.81 for the UCNP emission at 661 nm, and 0.89 for the other UCNP emission at 551 nm, respectively. The lower slope of this TH yield can be also explained by the reabsorption by thin-film UCNPs. The minor increase of the UCL yield is mainly due to the fact that the absorbed TH acts as a second excitation source for UCNPs, overcoming the small absorption cross-section area of UCNPs at 1,560 nm^20^.

### Radio-frequency domain analysis of the output emission from thin-film UCNPs

**Figure 3:**
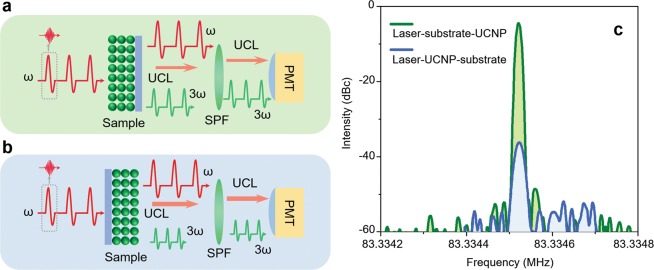
RF spectral analysis with two different excitation geometries. (**a,b**) Experimental systems for laser-UCNP-substrate and laser-substrate-UCNP geometries. (**c**) RF emission spectra from laser-UCNP-substrate and laser-substrate-UCNP geometries.

Because the TH and UCL are near to each other, it is difficult to analyze their contributions separately in the wavelength domain. Therefore, to confirm the TH contribution, RF domain analysis was additionally introduced here using a PMT as the high-speed photo-detector. A mode-locked femtosecond pulse laser emits a repetitive pulse train with the same pulse-to-pulse time spacing. The time spacing between the pulses in this investigation is 12 ns, which corresponds to 83.3 MHz in the pulse repetition rate. Note that the pulse repetition rate is the reciprocal of the time spacing. THG is a coherent photon conversion process having a time constant significantly shorter than a nanosecond, so it preserves the original repetition rate of the excitation femtosecond laser. Whereas, the UCL is governed by the relaxation time constant of UCNPs, which is longer than a microsecond. Therefore, the very ultra-short pulse duration and the high repetition rate of the excitation femtosecond laser cannot be preserved in UCL. By measuring the signal intensities at 83.3 MHz, the contribution of THG in the measured spectra can be identified without the interruption from the continuous UCL signal regardless their spectra overlap in the 530 nm region. For testing the repetition rates at different geometries, two experimental setups are configured as shown in Fig. 3a,b. A train of femtosecond pulses of 83.3 MHz repetition rate was focused onto the interface between the thin-film UCNPs and the substrate via the two sample geometries. The fundamental wavelength (1,560 nm) was filtered out using a short-pass filter (Thorlabs FES 1000) so as to detect the TH and UCL only without the background noise. A high-speed PMT with the radiant sensitivity of 78 mA/W was utilized to detect the pulse train with a 0.57 ns time resolution using an amplification factor of 10^6^. The detected electric signal was analyzed by an RF spectrum analyzer (Rigol DSA875) with a resolution bandwidth of 10 Hz from 83.333 to 83.335 MHz. Figure 3c shows the RF spectra of the PMT signals from the two geometries, laser-UCNP-substrate and laser-substrate-UCNP. The RF spectra from the laser-UCNP-substrate geometry clearly show a strong peak at the repetition rate with a signal-to-noise ratio higher than 50 dB, which indicates that the signal is more than 100,000 times stronger than the background noise. The repetition rate at TH matches well with the original repetition rate at the fundamental wavelength of the excitation femtosecond laser. These results confirm that the emission from thin-film UCNPs is dominated by TH; the TH contribution is 2,240 times stronger in laser-UCNP-substrate geometry than the contribution in laser-substrate-UCNP geometry, which can be identified by the 33.5 dB spectral power difference at the RF peaks as shown in Fig. 3c. This deviation comes from the difference in the excitation pathways in the two experiment geometries as explained in Section 2.

### Time-domain analysis of THG and UCNP emissions

**Figure 4:**
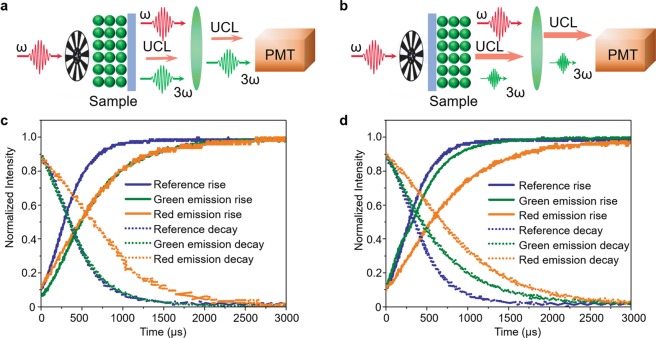
Time-domain analysis. (**a,b**) Experimental systems for measuring the rise and decay times at laser-UCNP-substrate and laser-substrate-UCNP geometries. (**c,d**) Rise and decay times for the emissions from laser-substrate-UCNP and laser-UCNP-substrate geometries.

To analyze the time response of UCL in more detail, time-domain analysis was carried out with a long time-span over 3,000 µs. The RF domain analysis in Section 3 is beneficial for testing TH characteristics requiring nanosecond-level high time resolution; however, it does not work well for UCL having a much longer time constant of over one microsecond. Figure 4a,b show the system configuration for the time-domain analysis. An optical chopper (Stanford Research Systems SR540) was additionally installed in front of the laser so as to modulate the intensity of the excitation laser beam at 100 Hz. A fiber-coupled continuous-wave laser diode at 635 nm wavelength was used as the reference laser for system characterization without UCNP samples. The time-domain analysis provided the rise and decay times which are shown in Fig. 4c,d. In the laser-UCNP-substrate geometry, the rise times of both green and red emissions to 50% from their maximum intensities are 190 μs, which indicates that UCNPs are mainly excited by the three-photon absorption process (See Fig. 4c). The green emission decays to 50% from its maximum in 30 μs, while the red emission does in 290 μs (See Fig. 4c); this faster decay of the green emission indicates that THG is significantly enhanced with the aid of thin-film UCNPs, and that THG plays the main role in the green emission peaked at 536 nm. It should be noted that the long rise time of the electron population at ^2^H_11/2_, resonant with TH, is mainly responsible for the slow rise of green emission. Although THG itself is an ultrafast phenomenon, the power amplification process inside UCNPs takes longer time due to the slow absorption process in UCNPs. Once the electron population at ^2^H_11/2_ becomes high enough for the power amplification, the time response is dominated by THG itself; thus, the pulse duration and repetition rate of the excitation pulses can be maintained thereafter. When UCNPs are excited in the laser-substrate-UCNP geometry, the rise time of the red emission is 290 μs, which is similar to the typical rise time of UCL excited by continuous-wave lasers. The rise time of the green emission around 551 nm is 60 μs, 230 μs faster than the UCL at 661 nm (See Fig. 4d). This shorter rise time of the green emission indicates that TH at 516 nm plays the major role in the excitation of UCL. THG, as an ultrafast phenomenon, excites the UCNPs via a single-photon process, which does not involve any intermediate energy level, results in the short rise time. Meanwhile, the UCL at red emission coming from the electron relaxation from ^4^F_9/2_ is governed by three-photon excitation process, which results in a long rise time. The decay times of green and red emissions are 90 and 340 μs, respectively. While the red emission at 661 nm follows with the decay time of UCL, the decay time of the green emission is relatively shorter due to the presence of remaining TH that is not absorbed by UCNPs.

### Power-amplified TH and UCNP emission from thin-film UCNPs on a single crystalline Si substrate

**Figure 5:**
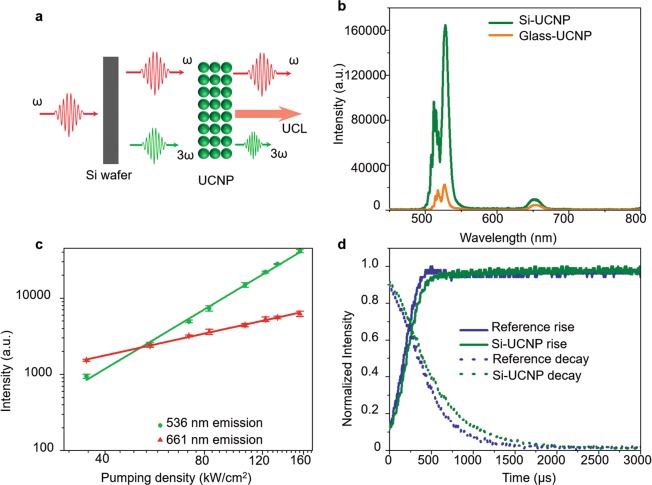
TH and UCL from the thin-film UCNPs deposited on Si substrate. (**a**) Schematics of TH amplification at thin-film UCNPs on a Si substrate. (**b**) Emission spectra of the thin-film UCNPs on top of Si and glass substrates. (**c**) Emission yield of the TH and UCL. (**d**) Rise and decay times of the TH and UCL.

A single crystalline Si wafer was tested as the second substrate for thin-film UCNPs instead of the glass as shown in Fig. 5a in order to study how the TH seed power affects to TH amplification in terms of the emission spectrum, yield strength, rise time, and decay time. Si wafers provide ~50 times higher THG efficiency than glass^1,26^. Besides, as a single crystalline material, Si wafer shows a clear crystalline orientation dependency in TH; this provides a deeper insight to the polarization coupling between TH and UCL, which will be dealt in detail in Section 6. The emission spectrum from the Si-UCNP is compared with the one from the glass-UCNP sample as shown in Fig. 5b. Note that the sample was excited only via the laser-substrate-UCNP geometry due to the limited spectral transmittance of Si; a 5.0-mm-thick Si has less than 0.00001% optical transmittance over the whole visible spectrum. Despite the 50-times higher THG efficiency at Si substrate, the intensity ratio between TH at 536 nm and UCL at 661 nm from thin-film UCNPs deposited on Si is only 2 times higher than the case supported by the glass substrate, which indicates the saturation of Er^3+^ absorption level at 520 nm in the thin-film UCNPs. As a result, the slope of UCNP emission peak at 536 nm is 2.70 as shown in Fig. 5c, 1.1 times higher than that of glass substrate coupled UCNPs. Highly doped UCNPs could provide higher TH energy without the gain saturation. The emission yield of UCL at 661 nm is 0.91, which is also slightly higher than the case with the glass substrate supported UCNPs (0.81). With a stronger green TH seed light from Si substrate, the rise time in the green band was shortened to 12 μs as shown in Fig. 5d, which is 18 μs shorter than that from the glass-supported thin-film UCNPs. The decay time was 20 μs with the Si substrate, which is also shorter than 150 μs, the decay time with the glass substrate. These shorter rise and decay times indicate that the major part of the green emission from the thin-film UNCPs on Si substrate comes from the TH after the reabsorption of UCL.

### Polarization-dependence in power-amplified TH and UCNP emission

**Figure 6:**
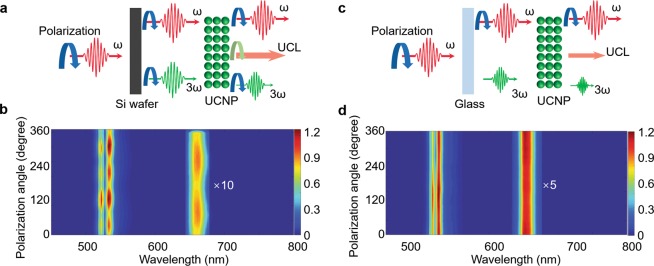
Power amplified TH and UCL under different incidence laser polarization state. (**a,c**) Experimental systems for characterizing the polarization dependence of the thin-film UCNPs supported by Si and glass substrates. (**b,d**) Two-dimensional maps of the spectral power distribution under different polarization states at the thin-film UCNPs supported by Si wafer and glass substrate.

The emission intensity dependence on input polarization state was finally tested (See Fig. 6a,c). While the input polarization was rotated from 0 to 360°, the TH and UCL spectra from thin-film UCNPs on Si and glass substrates were recorded and mapped into two-dimensional diagram as shown in Fig. 6b,d, respectively. Because Si is a face-centered crystalline material, the seed TH from the intrinsic (100) Si substrate provides a four-fold symmetry^1^. Following this polarization state of the seed TH, the spectral peak at 536 nm in Fig. 6b shows a clear four-fold symmetry. This result confirms that the spectral emission at 536 nm is from the amplified TH. UCNP emissions were reported to have a two-fold symmetry due to their non-uniform dipole orientations along the certain axes within the anisotropic nanocrystal^27^. The spectral UCL peak at 661 nm in Fig. 6b also shows clear two-fold symmetry. Different from the polarization dependence of TH from a bare Si wafer, the green spectral peaks from thin-film UCNPs on the Si substrate are more than 20% stronger at 120° and 300°. This spectral power redistribution is expected to come from the coupling between TH and UCL, which indicates that the power amplification of TH is more efficient when TH seed and UCL are stronger (like 120 and 300° in Fig. 6b). This can be explained by the alignment of the dipole orientation of UCNPs to the dipole in the Si crystal, which results in the resonance between UCNP absorption and TH emission. On the other hand, TH from the glass substrate is independent of the incident polarization state due to its amorphous nature. No clear polarization dependence was observed with the glass substrate as shown in Fig. 6d.

## Conclusion

To conclude, we demonstrated the coherent power amplification of TH femtosecond pulses by a normalized efficiency of 35,000% *W*^−1^ *cm*^−2^. at multi-layered thin-film UCNPs. This power amplification was enabled by the resonance of incident femtosecond laser field, its TH, and the electric bandgaps of UCNPs. The amplification factor was strongly dependent on the sample orientation; the TH emission yield was 3.5 in the laser-UCNP-substrate geometry and 2.5 in the laser-substrate-UCNP geometry. The RF spectral analysis at the pulse repetition rate of 83.3 MHz confirmed that TH portion is 2,240 times stronger in the laser-UCNP-substrate geometry than the laser-substrate-UCNP geometry. In addition, the laser-UCNP-substrate geometry also preserved the repetition rate and pulse duration better. Time-domain analysis revealed the power dominance of TH over UCL through the rise and decay times. By testing two different substrates, one amorphous glass and the other crystalline Si, seed power and polarization dependencies were revealed. The power amplification ratio at thin-film UCNPs can be maximized by coupling a stronger TH seed light, aligning the incident polarization state to the dipole orientation UCNPs, and optimizing the doping rate. This coherent amplification of TH at thin-film UCNPs provides higher power, shorter wavelength, and ultra-short pulse duration at simple thin-film geometry, which will contribute to realizing efficient test platform for material sciences, nano/micro integrated photonics, and biomedical diagnosis.

## Data Availability

The datasets generated during the current study are available from the corresponding author on reasonable request.
